# Quantitative analysis of vitamin E, 5-methyltetrahydrofolate, and glutathione in seminal plasma by liquid chromatography-tandem mass spectrometry

**DOI:** 10.3389/fchem.2026.1900122

**Published:** 2026-07-30

**Authors:** Tianwei Zhang, Bin Liu, Yang Fu, Yan Jin, Fang Luan, Yong Wang

**Affiliations:** 1 Department of Clinical Laboratory, Shandong Provincial Hospital Affiliated to Shandong First Medical University, Jinan, Shandong, China; 2 Department of Biomedical Engineering, Shandong Provincial Hospital Affiliated to Shandong First Medical University, Jinan, Shandong, China; 3 Department of Reproductive Medicine Center, Jinan Maternity and Child Care Hospital Affiliated to Shandong First Medical University, Jinan, Shandong, China

**Keywords:** 5-methyltetrahydrofolate, glutathione, liquid chromatography-tandem mass spectrometry (LC-MS/MS), male infertility, oxidative stress, vitamin E

## Abstract

**Background:**

Oxidative stress contributes to male infertility, but quantifying non-enzymatic antioxidants in seminal plasma remains challenging. This study aimed to establish liquid chromatography-tandem mass spectrometry (LC-MS/MS)-based methods for measuring vitamin E (VE), glutathione (GSH), and 5-methyltetrahydrofolate (5-MTHF) in human seminal plasma.

**Methods:**

Analyte-specific pretreatments were established: liquid–liquid extraction for VE, 96-well phospholipid removal plate for 5-MTHF, and sulfosalicylic acid precipitation for GSH without derivatization, with stable isotope-labeled internal standards. LC-MS/MS analysis was performed on AB SCIEX 4500 MD and Waters Xevo TQ-S systems. The method was validated in accordance with the CLSI C62-A guidelines. Preliminary method-specific reference intervals (RIs) were established in 120 healthy reproductive-aged men. Serum-seminal plasma correlations were assessed in 22 paired samples, and exploratory clinical associations were evaluated in 47 infertile men.

**Results:**

High linearity (R > 0.99) was achieved, with lower measuring interval limits of 78.10, 1.31, and 15.37 ng/mL for VE, 5-MTHF, and GSH, respectively. The intra- and inter-assay coefficients of variation were <6.8% and <5.5%, respectively. Recovery rates ranged from 88.78% to 108.10%. No significant matrix effects, carryover, or interference were observed. No significant seminal plasma-serum correlations detected. Seminal 5-MTHF and GSH levels were significantly lower in infertile men than in healthy controls [5-MTHF: 139.50 (68.02–198.50) vs. 387.80 (152.50–623.80) ng/mL, *P* < 0.001; GSH: 4314.00 (3051.00–5974.00) vs. 9049.00 (6128.00–11828.00) ng/mL, *P* < 0.001], whereas VE levels showed no significant difference [199.00 (138.00–252.00) vs. 168.50 (129.00–237.80) ng/mL, *P* = 0.211].

**Conclusion:**

The validated LC-MS/MS methods enable reliable quantification of seminal VE, 5-MTHF, and GSH and provide preliminary method-specific RIs in healthy Chinese men. Lower seminal 5-MTHF and GSH levels in infertile men suggest their potential relevance to male infertility. Further multicenter studies are needed to validate their clinical applicability.

## 1 Introduction

Male infertility is typically defined as the failure of a female partner to achieve a clinical pregnancy following at least 12 months of regular unprotected sexual intercourse due to male reproductive factors (Brannigan et al., 2024). Infertility affects approximately 15% of reproductive-aged couples worldwide, with male factors contributing to about 30%–50% of cases (Eisenberg et al., 2023). Although several etiologies, including varicocele, spermatogenic dysfunction, endocrine disorders, and genetic abnormalities, have been extensively investigated (Fang et al., 2021; Lamb, 2025; Sharma et al., 2021), the biological mechanisms underlying a considerable proportion of male infertility cases remain incompletely understood (Author anonymous, 2024). Among these mechanisms, oxidative stress (OS) has increasingly been recognized as a critical factor linked to male infertility (Evans et al., 2021; Minhas et al., 2025). Excessive reactive oxygen species (ROS) or insufficient antioxidant capacity in semen may impair sperm motility, promote lipid peroxidation, damage sperm DNA, and adversely affect embryo development (Ribeiro et al., 2022; Wright et al., 2014).

Seminal plasma provides the immediate biochemical microenvironment for spermatozoa and contains a complex network of antioxidant and metabolic factors. Within this network, vitamin E (VE), glutathione (GSH), and 5-methyltetrahydrofolate (5-MTHF) may reflect complementary aspects of a redox-epigenetic axis relevant to sperm function. VE is a lipid-soluble antioxidant that protects sperm membranes from lipid peroxidation (Su et al., 2022; Zhou et al., 2022); GSH is a major endogenous thiol antioxidant involved in redox buffering and ROS scavenging (Adeoye et al., 2018; Hamilton Lauren et al., 2022); and 5-MTHF, the biologically active form of folate, participates in one-carbon metabolism, homocysteine regulation, and DNA methylation, which maintains the stability of sperm genetic material (Boxmeer et al., 2009; Ferrazzi et al., 2020; Škovierová et al., 2016). Thus, accurate detection of these three non-enzymatic antioxidants in seminal plasma may provide an analytical basis for evaluating local antioxidant status and redox-related reproductive biology.

Reliable quantification of small-molecule antioxidants in seminal plasma remains technically challenging. Although liquid chromatography–tandem mass spectrometry (LC-MS/MS) methods have been reported for related analytes in blood-derived matrices (Fazili et al., 2013; Le et al., 2018; Lee et al., 2016; Li et al., 2026; Liu et al., 2022; Maxones et al., 2024; Moore et al., 2013; Wang et al., 2026), these methods cannot be directly applied to seminal plasma because of substantial differences in sample composition. Compared with serum or plasma, seminal plasma comprises viscous components, including high concentrations of seminal proteins, fructose, cellular debris, and mucopolysaccharides. This results in a matrix with high viscosity and pronounced heterogeneity, which is prone to matrix effects during pretreatment, and the sample stability is considerably lower than that of serum. In addition, the expected concentration ranges of VE, GSH, and 5-MTHF in seminal plasma may differ from those in blood, requiring matrix-adapted calibration, pretreatment, and validation procedures.

Therefore, the goals of this study were to develop and validate LC-MS/MS-based analytical procedures for accurate measurement of VE, GSH, and 5-MTHF levels specifically in seminal plasma. Following rigorous validation, this method was further utilized to establish preliminary method-specific reference intervals (RIs), filling the gap in current clinical detection technology and providing a methodological foundation for future studies investigating seminal antioxidant status in male infertility.

## 2 Materials and methods

### 2.1 Patients and samples

**FIGURE 1:**
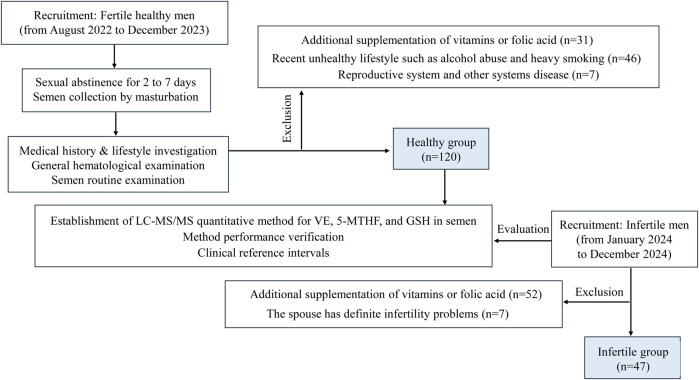
The participant enrollment flowchart.

Seminal plasma samples from 120 healthy reproductive-aged males and 47 males with infertility were collected from the Department of Reproductive Medicine Center, Jinan Maternity and Child Care Hospital Affiliated to Shandong First Medical University; additional serum samples were obtained from 22 of the healthy group. All subjects were enrolled strictly in accordance with the established inclusion and exclusion criteria, with no history of cardiac, hepatic, renal, or other systemic diseases, no use of vitamins, folic acid, lipoic acid, or other supplements that might affect the detection results within the preceding 3 months. Exclusion criteria for the healthy group should specifically exclude any reproductive system diseases. The inclusion criteria for the infertility group required that couples have regular unprotected sexual intercourse for at least 12 consecutive months without achieving a natural pregnancy, with no definite infertility-related problems in female partners. The study was approved by the Medical Ethics Committee of Jinan Maternity and Child Care Hospital in accordance with the Declaration of Helsinki (Approval No. 2020-1-033). The participant enrollment flowchart is presented in Figure 1.

Semen collection followed the guidelines of the World Health Organization Laboratory Manual for the Examination and Processing of Human Semen (Fifth Edition) (World Health Organization, 2010). All participants abstained from sexual activity for 2–7 days before sample collection. Semen was obtained via masturbation and collected in a specialized, wide-mouthed plastic container. A portion of the sample was used for routine semen analysis. The sample was then placed in a 37 °C constant-temperature water bath. After 30 min of liquefaction, routine semen analysis and detection of the DNA fragmentation index (DFI) were performed. The remaining sample was mixed with a commercial liquefying agent at a 100:1 ratio, then centrifuged at 4 °C. The liquefying agent used in this study was mainly composed of bromelain and sucrose. Bromelain, a broad-specificity proteolytic enzyme, was used to digest semen coagula and promote rapid liquefaction of viscous or incompletely liquefied samples under mild conditions. Because viscous semen was observed in many infertile men and in a small number of healthy participants, all samples were treated with the same liquefying agent at a fixed ratio to ensure uniform sample pretreatment. The supernatant was collected, immediately stored at −80 °C, and tested as soon as possible. Other serological biomarkers and semen parameters of the participants were assessed during routine clinical procedures and obtained from the laboratory information system.

### 2.2 Instruments, chemicals, and reagents

VE and 5-MTHF analyses were performed on an AB SCIEX Triple Quad™ 4500 MD equipped with a Jasper high-performance liquid chromatography (HPLC) system, using a Phenomenex C18 column (50 mm × 3.0 mm, 5 μm; Torrance, CA, USA) for chromatographic separation. Analyses of GSH were carried out on a Waters ACQUITY UPLC I-Class IVD/Xevo TQ-S system, using a Waters ACQUITY UPLC HSS T3 column (100 mm × 2.1 mm, 1.8 μm; Milford, MA, USA). The ultrapure water (≥ 18.0 MΩ.cm, 25 °C) used in the experiment was prepared by a Milli-Q ultrapure water meter (Millipore Corporation, USA). Other experimental consumables included a Waters Ostro™ 96-well phospholipid removal plate and a 96-well 350 μL ACQUITY collection plate (Milford, MA, USA).

HPLC-grade methanol, acetonitrile, formic acid (FA), hexane, ammonium formate, and isopropanol were purchased from Merck Millipore (Billerica, MA, USA). Both 5-sulfosalicylic acid and ascorbic acid were purchased from Mass Spectrometry Biotechnology Co., Ltd. (Yantai, Shandong, China). VE, methyltetrahydrofolic acid disodium salt, and GSH calibrators were purchased from Sigma-Aldrich (Saint Louis, MO, USA). Stable isotope-labeled internal standards (ISs) were used for LC-MS/MS quantification to correct potential variations during sample preparation, chromatographic separation, ionization, and detection. Methyltetrahydrofolic acid (Glutamic acid-^13^C_5_) IS was procured from Cambridge Isotope Laboratories, Inc. (Cambridge, MA, USA). Vitamin E-d_6_ IS was obtained from SHANGHAI ZZBIO CO., LTD. (Shanghai, China). Additionally, GSH (glycine-^13^C_2_,^15^N) IS was purchased from Shanghai Pufen Biotechnology Co., LTD. (Shanghai, China). Semen liquefying agent was purchased from Shenzhen Boruide Biotechnology Co., Ltd. (Shenzhen, Guangdong, China).

### 2.3 Sample preparation

#### 2.3.1 Preparation of calibrator and IS solutions

The powders of VE, 5-MTHF, and GSH calibrators were each dissolved in 50% methanol–water containing 0.1% bovine serum albumin (BSA) to prepare stock solutions at concentrations of 1 × 10^3^ μg/mL, 65.83 μg/mL, and 4 × 10^4^ μg/mL, respectively. The stock solutions were then serially diluted with 50% methanol–water (0.1% BSA) to prepare calibration working solutions. The concentrations of the VE working solutions were 78.1, 156, 313, 625, 1250, 2500, 5000, 10,000, and 20,000 ng/mL. The concentrations of the 5-MTHF working solutions were 1.31, 3.48, 7.72, 16.13, 32.89, and 65.83 ng/mL. The concentrations of the GSH working solutions were 15.37, 30.73, 153.66, 307.32, 1536.60, and 3073.20 ng/mL. The powders of the corresponding stable isotope-labeled ISs were also dissolved in 50% methanol–water to prepare IS stock solutions. The concentrations of the IS working solutions were as follows: VE, 5 μg/mL; 5-MTHF, 37.15 μg/mL; and GSH, 1.25 μg/mL. All prepared solutions were aliquoted and stored at −80 °C to avoid freeze–thaw cycles to preserve stability.

#### 2.3.2 Preparation of samples

VE sample preparation (protect from light): A total of 200 μL of the sample (calibrator working solution or seminal plasma) was mixed with 6 μL of IS working solution and 300 μL of acetonitrile and then vortexed for 5 min. Then, a total of 1400 μL of hexane was added to the mixture and shaken for 5 min. After centrifugation for 5 min at 12,000 g and 4 °C, the extraction supernatant was transferred to a deep 96-well plate and dried with N2. Then, a total of 100 μL methanol:acetonitrile:isopropanol (4:4:2; v/v/v) was added to the sample and vortexed.

5-MTHF sample preparation (protect from light): A total of 120 μL of the sample (calibrator working solution or seminal plasma) was mixed with 20 μL of IS working solution and 460 μL of methanol containing 0.1% ascorbic acid. The mixture was vortexed for 5 min. After centrifugation at 12,000 g and 4 °C for 5 min, 500 μL of the supernatant was transferred to 96-well phospholipid removal plate. The filtrate was collected in the 96-well collection plate and dried with N_2_. Then, a total of 50 μL ddH_2_O containing 0.1% ascorbic acid was added to the sample and vortexed.

GSH sample preparation (protect from light): A total of 200 μL of the sample (calibrator working solution or seminal plasma) was mixed with 20 μL of IS working solution and 120 μL of extractant (main components: 5-sulfosalicylic acid, isopropanol, and ascorbic acid). The mixture was vortexed for 10 min. After centrifugation at 14,000 g and 4 °C for 20 min, 100 μL of the supernatant was transferred to a deep 96-well plate.

### 2.4 LC-MS/MS procedure

All three analyte profiles were based on electrospray ionization-mass spectrometry (ESI-MS) data combined with tandem mass spectrometry. Quantitation by multiple reaction monitoring (MRM) analysis was performed in the positive ion mode. Only one optimized quantifier MRM transition was retained for each analyte in the final clinical quantitative method. Additional product ion transitions were evaluated during method development and preliminary optimization but were not used in the final routine quantitative workflow. The specificity of the selected transitions was further supported by chromatographic separation, stable isotope-labeled ISs, and method validation experiments. The injection volumes were selected as the minimum volumes that provided adequate sensitivity while minimizing the amount of residual seminal plasma matrix introduced into the LC-MS/MS system, thereby reducing potential matrix effects. The elution gradients and mobile phases for all three analyses were shown in Table 1; LC-MS/MS systems, analytical columns, injection volumes, retention times, and MS/MS parameters, are summarized in Table 2.

**TABLE 1:** The elution conditions of VE, 5-MTHF and GSH for the LC separation.

Analyte	Mobile phase A	Mobile phase B	Step	Time (min)	Flow (mL/min)	A (%)	B (%)	B. Curve
VE	Ultrapure water with 0.1% FA	Methanol with 5 mM ammonium formate	1	0.0	0.6	35	65	0
			2	0.1	0.6	35	65	0
			3	1.5	0.6	2	98	0
			4	5.5	0.6	2	98	0
			5	5.6	0.6	35	65	0
			6	5.61	0.6	35	65	0
5-MTHF	Ultrapure water with 0.3% FA	methanol	1	0.0	0.5	95	5	0
			2	1.0	0.5	95	5	0
			3	1.2	0.5	50	50	0
			4	1.8	0.5	30	70	0
			5	2.5	0.5	5	95	0
			6	3.5	0.5	5	95	0
			7	3.51	0.5	95	5	0
GSH	Ultrapure water with 0.1% FA	acetonitrile	1	0.0	0.3	98	2	0
			2	1.0	0.3	98	2	6
			3	1.5	0.3	40	60	6
			4	2.0	0.3	1	99	6
			5	3.0	0.3	1	99	1
			6	5.0	0.3	98	2	1

A (%) and B (%) indicate the proportions of mobile phase A and mobile phase B, respectively; B. Curve represents the gradient curve setting used in the LC method.

**TABLE 2:** Analyte-specific LC-MS/MS systems, chromatographic conditions, and MS/MS parameters.

Parameter	VE	5-MTHF	GSH
LC-MS/MS system	AB SCIEX Triple Quad™ 4500 MD with Jasper HPLC system	AB SCIEX Triple Quad™ 4500 MD with Jasper HPLC system	Waters ACQUITY UPLC I-Class IVD/Xevo TQ-S system
Column temperature (°C)	45	40	40
Autosampler temperature (°C)	10	10	10
Injection volume (μL)	15	3	2
Retention time (min)	3.90	2.48	1.28
Mode	ESI+	ESI+	ESI+
Quantifier mass transition-target analyte (m/z)	431.4 > 165.3	460 > 313	308.1 > 178.9
Quantifier mass transition-internal standard (m/z)	437.4 > 171.3	464 > 313	311 > 181.9
Dwell time (ms)	15	40	15
Collision energy (V)	30	30	10
Instrument-specific ion-path parameters	Declustering potential, 36 V; entrance potential, 10 V; collision cell exit potential, 3 V	Declustering potential, 60 V; entrance potential, 10 V; collision cell exit potential, 6 V	Cone voltage, 30 V; capillary voltage, 3.4 kV
Instrument-specific ion-source parameters	CUR, 25 psi; CID, 6 psi; IonSpray voltage, 5500 V; GS1, 80 psi; GS2, 75 psi	CUR, 10 psi; CID, 7 psi; IonSpray voltage, 5500 V; GS1, 70 psi; GS2, 70 psi	Desolvation gas flow rate, 1200 L/h; cone gas flow rate, 150 L/h
Source temperature (°C)	500	500	500

CUR, curtain gas; CID, collision-induced dissociation; GS, ion source gas.

### 2.5 Method validation

To validate the developed LC-MS/MS assay, parameters including linearity, limit of detection (LoD), lower limit of measuring interval (LLMI), specificity, matrix effect, accuracy (recovery), precision (intra-assay/inter-assay), carryover, interference, sample stability, and preliminary method-specific RIs were evaluated in accordance with the Clinical and Laboratory Standards Institute (CLSI) guideline C62-A (CLSI, 2014) and C50-A (CLSI, 2007) for bioanalytical method validation.

#### 2.5.1 Linearity, LoD, and LLMI

The linearity of the assay for VE, 5-MTHF, and GSH was assessed across the calibration range using regression analysis. LoD refers to the lowest concentration of analytes that can be detected under the established LC-MS/MS conditions, and it was used only to support the determination of the LLMI. The signal-to-noise ratio was at least 3:1. LLMI refers to the lowest detectable value that meets requirements for accuracy and precision of the laboratory. Samples with analyte concentrations near LoD were measured for 10 times. Criteria for LLMI included a mean deviation within 15% and the coefficient of variation (CV) < 20%.

$$\text{Recovery}\left(\%\right)=\frac{\text{Average}\;\text{measured}\;\text{concentration}}{\text{Spiked}\;\text{concentration}}\times 100\%$$

$$\text{CV}\left(\%\right)=\frac{\text{Standard}\;\text{deviation}}{\text{Mean}\;\text{measured}\;\text{concentration}}\times 100\%$$

#### 2.5.2 Specificity and matrix effect

Specificity was evaluated using double-blank samples (without IS or analytes), five LLMI-level samples, and five samples containing only IS. The ratios of the background peak area to the analyte peak area and to the IS peak area were calculated to assess specificity. The matrix effect was evaluated using a matrix-mixing experiment (El-Khoury et al., 2012). Three seminal plasma samples were selected and mixed with standard solutions at volume ratios of 0:100, 50:50, and 100:0 to prepare pure standard solution, 50% sample-standard mixture, and pure seminal plasma sample, respectively. The same volume of IS was then added to each sample, followed by identical sample pretreatment and LC-MS/MS analysis. The peak area ratios of the analyte to the IS in the pure standard solution, 50% mixed solution, and pure seminal plasma sample were recorded as A, C, and E, respectively. The relative matrix effect of the 50% mixed solution was calculated using the following formula:

$$\text{Relative matrix effect}\left(\%\right)=\frac{C-\left(0.5A+0.5E\right)}{0.5A+0.5E}\times 100\%$$

#### 2.5.3 Accuracy and recovery

The accuracy was evaluated by recovery. Three levels of calibrators with known concentrations were spiked into seminal plasma samples. VE, 5-MTHF, and GSH were each tested 5 times at each concentration. Recovery was calculated as the ratio of measured concentration to expected concentration.

#### 2.5.4 Precision (intra-assay and inter-assay)

Precision was evaluated using intra-assay precision and inter-assay variability, with four replicates measured over five consecutive days. For VE and GSH, three concentration levels (low, medium, and high) were analyzed. For 5-MTHF, two concentrations (low and high) were tested. These concentrations spanned the entire calibration curve range. The CVs were calculated to assess the precision.

#### 2.5.5 Carryover

After sample pre-processing, the lowest concentration of the standard curve was repeatedly injected, followed by the highest and lowest concentrations of the standard curve, alternately. The peak area ratios of the analyte to the IS were recorded as L1, L2, H, and L3, respectively.

$$\text{Carryover}=\frac{L3-L2}{H-L2}\times 100\%$$

#### 2.5.6 Interference

To assess potential interference from the liquefaction reagent used in semen processing, semen samples at three concentration levels (low, medium, and high) were prepared under three conditions, respectively, including: (1) without liquefaction reagent, (2) with reagent diluted 1:100, and (3) with reagent diluted 1:10. The relative deviation in quantitative results among the groups, calculated as (actual concentration–baseline concentration)/baseline concentration, was analyzed to evaluate interference.

#### 2.5.7 Sample stability

Three different concentrations (low, medium, and high) of semen samples were stored at −80 °C for 1 week, 1 month, and 6 months, respectively. The relative deviation was analyzed to determine the sample stability over time.

### 2.6 Statistical analysis

All data were analyzed using GraphPad Prism software version 9.0 (La Jolla, CA, USA) and SPSS 26.0 statistical software (IBM Corporation, USA). Data distribution normality was evaluated using the Shapiro-Wilk test. Normally distributed quantitative data were expressed as mean ± standard deviation (SD), and comparisons between groups were performed using the t-test. Non-normally distributed quantitative data were presented as median (P25–P75) and compared using the Mann-Whitney U test. Multivariate linear regression and binary logistic regression models were established to evaluate the stability of RIs and to analyze correlations among analytes, infertility status, and semen parameters. Linear equations and Pearson’s correlation analysis were used to examine correlations between quantitative variables. Preliminary method-specific RIs were established according to the CLSI guideline EP28-A3c (2008) (CLSI, 2010), with 95% confidence intervals (P2.5–P97.5) for non-parametric data.

All statistical tests were two-tailed, with *P* < 0.05 considered statistically significant.

## 3 Results

### 3.1 Evaluation of assay performance

#### 3.1.1 Linearity, LoD, and LLMI

**FIGURE 2:**
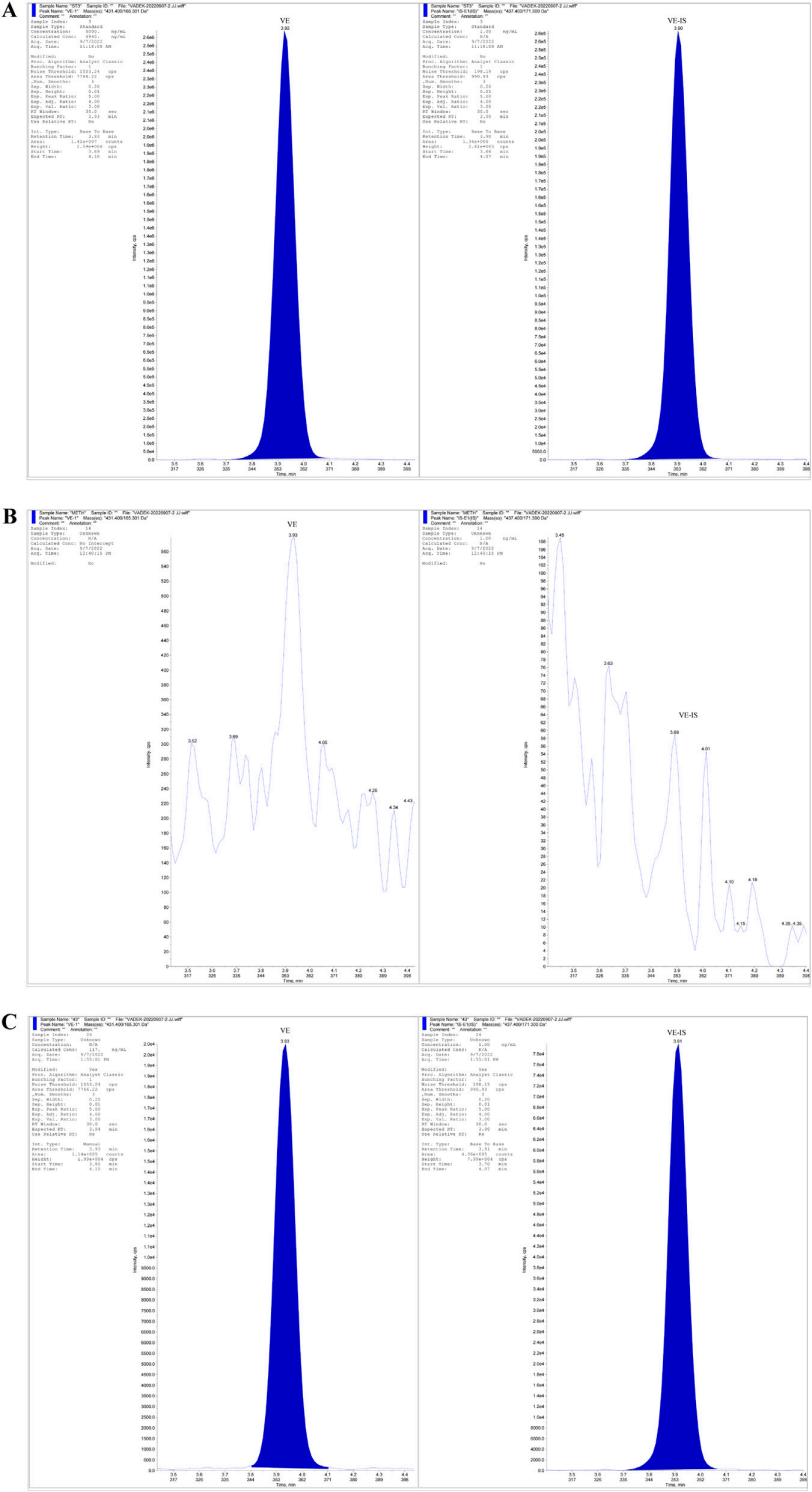
Representative chromatograms of standards **(A)**, double blanks **(B)** and seminal plasma sample **(C)** for VE.

**FIGURE 3:**
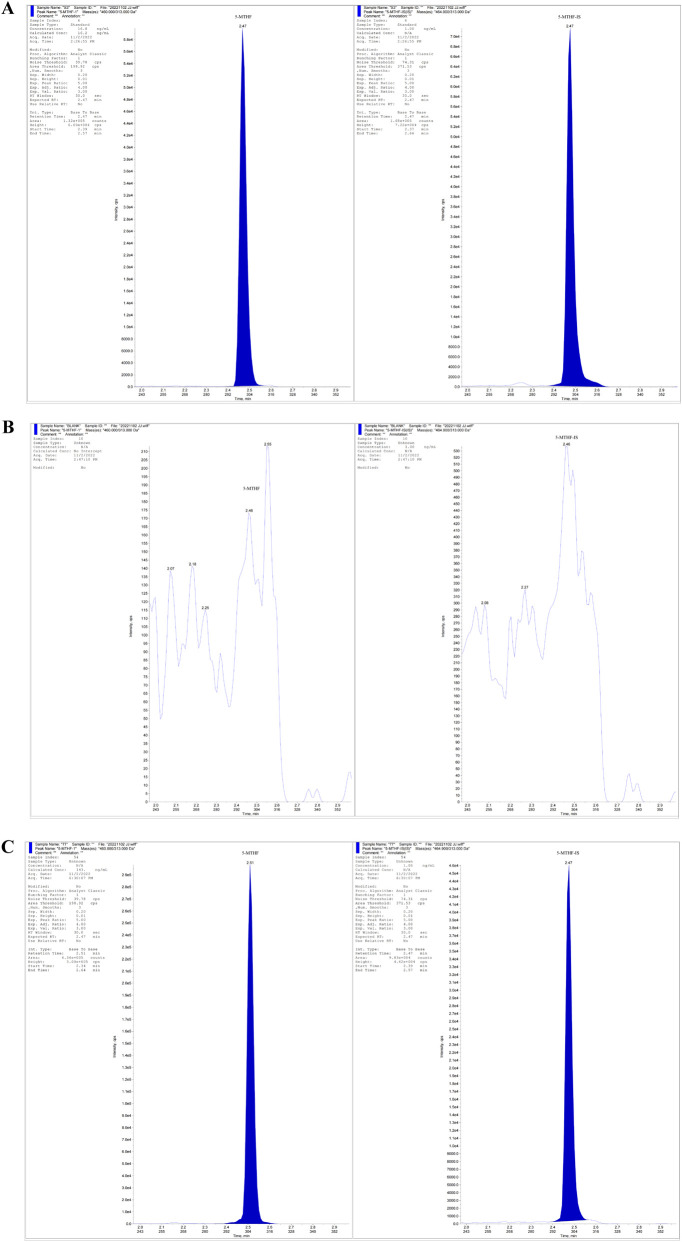
Representative chromatograms of standards **(A)**, double blanks **(B)** and seminal plasma sample **(C)** for 5-MTHF.

**FIGURE 4:**
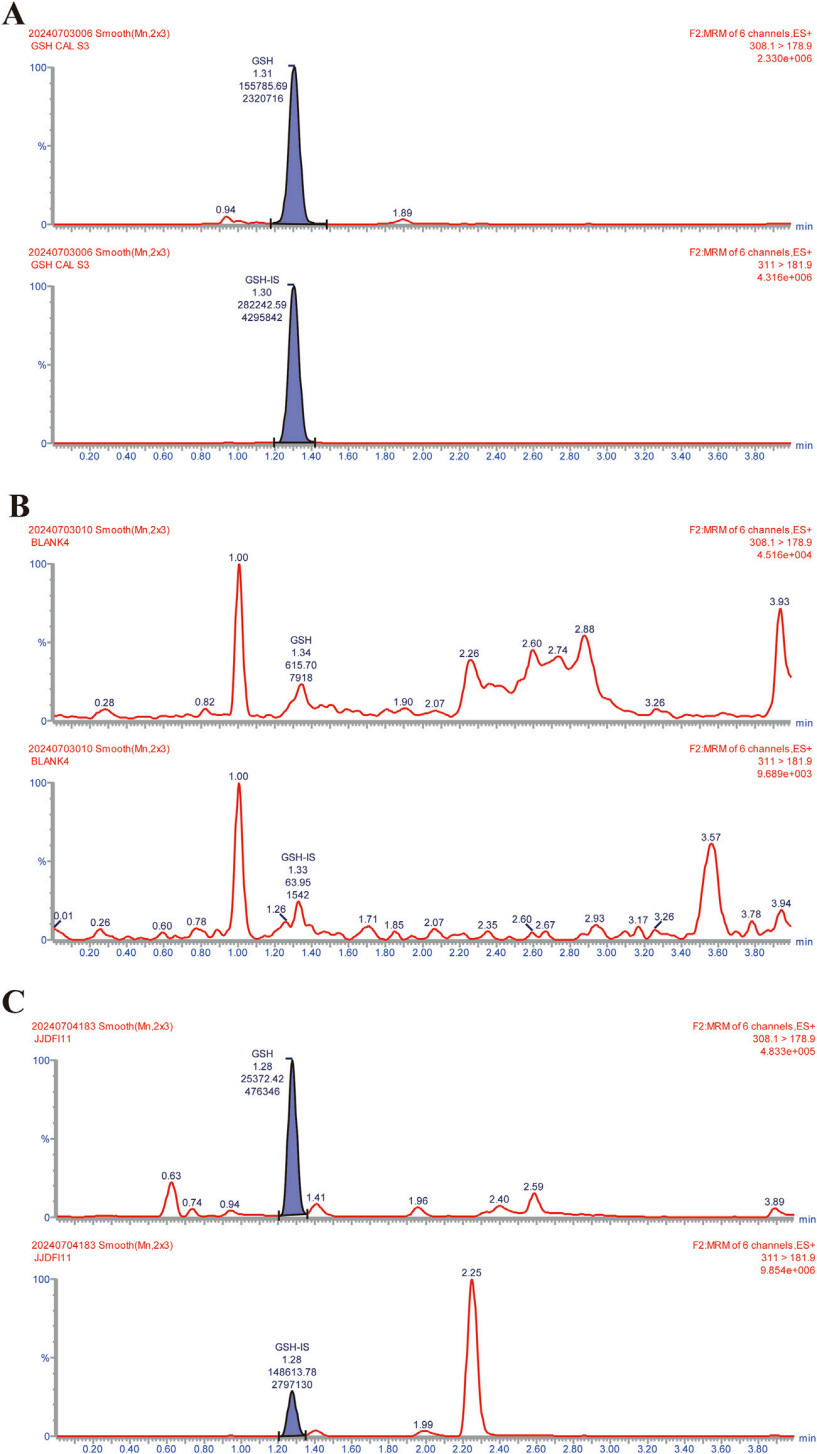
Representative chromatograms of standards **(A)**, double blanks **(B)** and seminal plasma sample **(C)** for GSH.

Representative chromatograms for VE, 5-MTHF and GSH are displayed in Figures 2–4, respectively, including standards, double blanks, and seminal plasma samples. For each analyte, calibration standards were prepared and analyzed in ten independent analytical batches. One calibration curve was generated in each batch, and the linearity parameters obtained from the ten calibration curves were summarized in Supplementary Table S1. The R values of the linearity regression analysis were all >0.99 and ranged from 0.996 to 0.999 for VE, 0.994 to 0.999 for 5-MTHF, and 0.998 to 0.999 for GSH (Table 3). The lowest concentration that met the CV criterion (< 15%) was determined as the LLMI for VE (78.1 ng/mL), 5-MTHF (1.31 ng/mL), and GSH (15.37 ng/mL) (Table 3).

**TABLE 3:** The calibration range, LLMI, and LoD of VE, 5-MTHF and GSH.

Analyte	Calibration range (ng/mL)	r value	LLMI (ng/mL)	CV at LLMI (%, n = 10)	Recovery at LLMI (%, n = 10)	LoD (ng/mL)
VE	78.10-20000	0.996∼0.999	78.10	4.64	102.37% (±4.39%)	39.05
5-MTHF	1.31-65.83	0.994∼0.999	1.31	4.34	97.82% (±4.12%)	1.31
GSH	15.37-3073	0.998∼0.999	15.37	4.41	103.39% (±4.19%)	15.37

LLMI, lower limit of measuring interval; LoD, limit of detection; CV, coefficient of Variation; IS, internal standard; VE, vitamin E; 5-MTHF, 5-methyltetrahydrofolate; GSH, glutathione.

#### 3.1.2 Specificity and matrix effect

For all three analytes, the background peak area/LLMI peak area was <1.5%, and the background peak area/IS peak area was <0.2% (Table 4). These data indicated that this LC-MS/MS method for the determination of VE, 5-MTHF, and GSH exhibited no obvious background interference. The matrix effect validation results showed that the relative matrix effect ranged from −11.98% to −0.93% for VE, from −1.56% to 10.33% for 5-MTHF, and from 1.01% to 1.24% for GSH. None of the three levels of concentration (low, middle, and high) of VE, 5-MTHF, and GSH showed a significant promoting or inhibitory effect (Supplementary Table S2).

**TABLE 4:** The specificity analysis for VE, 5-MTHF and GSH.

Analyte	No.	Double-blank area	LLMI area	Ratio (%)	Double-blank area	IS area	Ratio (%)
VE	1	2.55E+01	1.60E+05	0.02	5.35E+01	1.44E+06	0.00
	2	2.04E+01	1.60E+05	0.01	6.54E+01	1.49E+06	0.00
	3	8.68E+01	1.74E+05	0.05	8.72E+01	1.51E+06	0.01
	4	6.35E+01	8.13E+04	0.08	5.33E+01	6.25E+05	0.01
	5	1.80E+01	8.06E+04	0.02	5.06E+01	6.28E+05	0.01
5-MTHF	1	2.10E+02	3.24E+04	0.65	2.63E+02	1.43E+05	0.18
	2	2.20E+02	5.06E+04	0.43	2.13E+02	2.35E+05	0.09
	3	1.31E+02	4.90E+04	0.27	1.43E+02	2.15E+05	0.07
	4	2.48E+02	5.26E+04	0.47	3.70E+02	2.29E+05	0.16
	5	1.31E+02	4.47E+04	0.29	1.25E+02	1.99E+05	0.06
GSH	1	7.30E+01	1.35E+04	0.54	6.20E+01	2.90E+05	0.02
	2	1.73E+02	1.54E+04	1.13	1.27E+02	3.65E+05	0.03
	3	1.22E+02	1.60E+04	0.76	2.01E+02	3.66E+05	0.05
	4	9.40E+01	1.64E+04	0.57	6.90E+01	3.64E+05	0.02
	5	2.03E+02	1.54E+04	1.32	9.00E+01	3.63E+05	0.02

LLMI, lower limit of measuring interval; IS, internal standard; VE, vitamin E; 5-MTHF, 5-methyltetrahydrofolate; GSH, glutathione.

#### 3.1.3 Accuracy

Three levels of calibrators with different concentrations were added to the seminal plasma samples, respectively. VE, 5-MTHF, and GSH were tested 5 times at each level, respectively. The ratio of the measured concentration to the expected concentration was calculated and used as recovery indicator. The recovery rates ranged from 88.78% to 108.10%, with all deviations below 15%, which were considered acceptable (Table 5).

**TABLE 5:** The accuracy analysis for VE, 5-MTHF and GSH.

Analyte	Level	Three levels of calibrators (ng/mL)	Seminal plasma samples (ng/mL)	Theoretical value (ng/mL)	Measured value (ng/mL)	Recovery (%)
VE	Low	98.43	508.50	303.46	290.00	95.56
	Middle	151.00	508.50	329.75	292.75	88.78
	High	372.75	508.50	440.63	411.50	93.39
5-MTHF	Low	3.53	65.83	34.68	33.24	95.85
	Middle	14.43	65.83	40.13	40.29	100.40
	High	68.46	65.83	67.15	69.15	102.99
GSH	Low	338.11	774.57	556.34	582.70	104.74
	Middle	753.06	774.57	763.81	734.38	96.15
	High	1355.50	774.57	1065.04	1151.33	108.10

VE, vitamin E; 5-MTHF, 5-methyltetrahydrofolate; GSH, glutathione.

#### 3.1.4 Intra-assay precision and inter-assay precision analyses

The intra-assay precision was evaluated by analyzing samples at different concentration levels in four replicates within a single run. The CVs for all analytes ranged from 0.7% to 6.8%, all of which were below the predefined acceptance criterion (15%). The inter-assay CVs ranged from 2.3% to 5.5%, also within the acceptable limit of 15%. These results demonstrated that the developed LC-MS/MS method provided satisfactory precision and reproducibility for the quantification of the target biomarkers across the analytical ranges (Supplementary Table S3).

#### 3.1.5 Carryover

The carryover rates of VE, 5-MTHF, and GSH reached 0.01%, 0.00%, and 0.16%, respectively. The peak area of the blank sample injected after the highest calibration standard was 20% lower than that of the lowest calibration standard. These results indicated that there was no evident carryover.

#### 3.1.6 Interference

Three different concentrations (low, medium, and high) of semen with different proportions or without liquefying agent were tested. Compared to the quantitative analysis without the addition of the liquefying agent, the relative deviation of quantitative analysis was all <15% for VE, 5-MTHF, and GSH (Supplementary Table S4).

#### 3.1.7 Sample stability

Three different concentrations (low, medium, and high) of frozen semen (−80 °C) were assayed at 1 week, 1 month, and 6 months. Compared with the quantitative analysis of the fresh semen, the deviation of quantitative analysis was all <15% for at for VE, 5-MTHF, and GSH (Supplementary Table S5).

### 3.2 Sample dilution verification

The same linearity analysis of 5-MTHF was applied to serum and seminal plasma samples, with 5 times pre-dilution for seminal plasma samples. Dilution was performed using the original, unprocessed seminal plasma before IS addition and before sample preparation. The measured concentration was then multiplied by the corresponding dilution factor to obtain the final concentration in the original sample. For samples that remained above the upper limit of the calibration range after routine dilution, additional dilution verification was performed using the original unprocessed samples. For samples with concentrations below 3 times the LoD after 5-fold dilution, undiluted sample aliquots were reanalyzed to verify low-concentration quantification. The relative deviations were within the predefined acceptable ranges (Supplementary Table S6).

Similarly, the same linearity was employed for measuring the GSH content in serum and seminal plasma samples. The seminal plasma samples required a 5-fold dilution. For samples that remain above the linear upper limit after dilution, additional dilution verification was performed using original unprocessed sample. The deviation of quantitative analysis is from −14.01% to 9.88%. (Supplementary Table S7).

### 3.3 Comparison with previously reported LC-MS/MS methods

To further evaluate the analytical performance of the developed LC-MS/MS method, previously reported LC-MS/MS methods for VE, 5-MTHF, and GSH were compared with the present study in terms of sample matrix, sample volume, sample pretreatment, IS, calibration range, LoD/LLMI/LLOQ, precision, accuracy, specificity, and matrix effect (Table 6). Most reported methods were established using serum or whole blood as the biological matrix, whereas the present method was optimized for seminal plasma.

**TABLE 6:** Comparison with previously reported LC-MS/MS methods.

References	Target analyte	Matrix	Sample(μL)	Sample pretreatment	IS	Calibration range (ng/mL)	LoD/LLMI/LLOQ (ng/mL)	Precision (%)	Accuracy (%)	Specificity (%)	Matrix effect (%)	RIs (ng/mL)
Le et al. (2018)	VE	Amniotic fluid	60	One-step protein precipitation with ACN containing SIL-IS	Vitamin E-d6	10-500	10 (LLOQ)	4.50–9.10 (intra) 4.80–7.30 (inter)	87-107	85–115	88.4-112.6	—
Li et al. (2026)		serum	100	SALLE	Vitamin E-d6	625-40000	313 (LoD) 625 (LLOQ)	2.94–6.85 (intra) 2.97–5.76 (inter)	97.11-101.91	0.35–3.10 (double-blank)	86.56-98.55	—
Present study		Seminal plasma	200	LLE	Vitamin E-d6	78.10-20000	39.05 (LoD) 78.10 (LLMI)	2-4.40 (intra) 5.30–5.50 (inter)	88.78-95.56	0.00–0.08 (double-blank)	88.02-99.07	61.40–401
Fazili et al. (2013)	5-MTHF	Serum	150	96-probe SPE system	5-MTHF-^13^C_5_	0-45.95	0.03 (LoD) 0.09 (LLOQ)	1.90–2.50 (intra) 2.20–3.40 (inter)	93-105	Interference from other folate forms <0.01%	Serum vs. water calibration slope difference 4.4%	—
Liu et al. (2022)		Serum	100	SPE	5-MTHF-^13^C_5_	—	—	1.47–2.87 (intra) 1.71–2.77 (inter)	96.30-109.30	—	—	1.75-28.64
Present study		Seminal plasma	120	PPT + phospholipid-removal plate cleanup	5-MTHF-^13^C_5_	1.31-65.83	1.31 (LoD) 1.31 (LLMI)	6.40–6.80 (intra) 3.10–3.70 (inter)	95.85-102.99	0.06–0.65 (double-blank)	98.44-110.33	51.60–2010
Moore et al. (2013)	GSH	Whole blood	50	Derivatization	GSH- ^13^C	7683–153660	122.93 (LoD) 461 (LLOQ)	3.30 (intra) 4.10 (inter)	95-97	—	—	2.77 × 105 ± 43,024.80 (mean ± SD)
Lee et al. (2016)		Whole blood (mice)	60	Derivatization	GSH 13C^2^, 15N	30,732 – 1536605	15,366.50 (LoD) 23,049.75 (LLMI)	2-4.30 (intra) 4.10 (inter)	98-105.90	—	104.5	—
Present study		Seminal plasma	200	sulfosalicylic acid precipitation	GSH 13C^2^, 15N	15.37-3073	15.37 (LoD) 15.37 (LLMI)	0.70–2.10 (intra) 2.30–3 (inter)	96.15-108.10	0.02–1.32(double-blank)	101.01-101.24	3411.04–29833.33

IS, internal standard; LLMI, lower limit of measuring interval; LoD, limit of detection; LLOQ, lower Limit of Quantification; ACN, acetonitrile; SALLE, Salting-Out Assisted Liquid-Liquid Extraction; LLE, liquid-liquid extraction; SPE, Solid Phase Extraction; PPT, protein precipitation; VE, vitamin E; 5-MTHF, 5-methyltetrahydrofolate; GSH, glutathione.

Overall, compared with previously reported LC-MS/MS methods, the developed method exhibited acceptable assay performance, indicating that it is suitable for the quantitative analysis of VE, 5-MTHF, and GSH in seminal plasma.

### 3.4 Preliminary method-specific RIs *for healthy* reproductive-aged *males*

The baseline characteristics of the 120 healthy reproductive-aged males included in this study were summarized in Table 7. The baseline data were relatively homogeneous, indicating that the study population was suitable for establishing preliminary method-specific RIs for seminal VE, 5-MTHF, and GSH measured by LC-MS/MS. Based on these 120 healthy reproductive-aged men, the RIs of VE, 5-MTHF, and GSH in seminal plasma were determined. The reference ranges of VE, 5-MTHF, and GSH were 61.40–401, 51.60–2010, and 3411.04–29833.33 ng/mL, respectively. Histogram and box plot of the preliminary method-specific RIs distribution are shown in Supplementary Figure S1.

**TABLE 7:** The baseline characteristics of the participants.

Variables	Healthy group (n = 120)	Infertility group (n = 47)	t/Z value	*P* value
Age (yr)	28 (24,31)	32 (29, 34)	5.428	<0.001
Hemoglobin (g/L)	156.30 ± 9.19	153 ± 12.28	1.885	0.061
ALT (U/L)	24.50 (17.25, 38.75)	27 (22, 35)	0.612	0.540
TP (g/L)	75.88 ± 4.30	76.41 ± 5.20	0.682	0.496
ALB (g/L)	46.80 (45.50, 48.38)	46.70 (44, 49.80)	0.875	0.381
GLB (g/L)	28.90 ± 4.79	29.74 ± 6.76	0.908	0.365
GLU (mmol/L)	4.89 ± 0.50	5.05 ± 0.88	1.525	0.129
BUN (mmol/L)	5.61 ± 1.05	5.86 ± 1.51	1.211	0.228
CR (μmol/L)	74.15 ± 10.20	77.79 ± 13.60	1.879	0.062
SV (mL)	3.60 (2.73, 4.70)	4 (3.10, 5.60)	1.900	0.057
SC (10^6/mL)	58.34 (36.30)	71.66 (37.99, 115.30)	1.603	0.109
PR (%)	56.19 (44.65, 67.01)	38.50 (23.49, 49.55)	6.080	<0.001
TSM (%)	65.91 (52.02, 75.96)	48.58 (31.10, 58.85)	5.201	<0.001
DFI (%)	11.74 (8.75, 14.93)	20.33 (16.64, 28.29)	7.470	<0.001

ALT, alanine aminotransferase; TP, total protein; ALB, albumin; GLB, globulin; GLU, glucose; BUN, urea nitrogen; CR, creatinine; SV, semen volume; SC, sperm concentration; PR, progressive motility sperm; TSM, total sperm motility; DFI, DNA fragmentation index.

To further evaluate potential influencing factors, multiple linear regression analyses were performed using ln-transformed VE, 5-MTHF, and GSH as dependent variables, respectively. Based on the principle of avoiding model overfitting and multicollinearity, we selected representative variables from different clinical domains as adjustment factors, including liver function indicators such as alanine aminotransferase (ALT) and total protein (TP), the renal function indicator creatinine (CR), the metabolic indicator globulin (GLU), and semen parameters including sperm concentration (SC), progressive motility (PR), and DFI as independent variables. As shown in Supplementary Table S8, none of the included variables was significantly associated with lnVE, ln5-MTHF, or lnGSH concentrations in the healthy group, with all regression coefficients having *P* values >0.05. In addition, the overall ANOVA tests of the regression models were also not statistically significant. The VIF values ranged from 1.036 to 3.909, indicating no obvious multicollinearity among the independent variables. These findings suggested that the established RIs were not substantially affected by the investigated covariates in this healthy population, supporting the relative robustness of the preliminary method-specific RIs. Therefore, no further stratification or covariate adjustment was applied when establishing the preliminary method-specific RIs.

### 3.5 Consistency analysis of VE, 5-MTHF, and GSH levels in seminal plasma and serum

To explore the consistency and potential correlation of VE, 5-MTHF, and GSH levels between seminal plasma and serum, a total of 22 paired samples from the healthy group were simultaneously detected using the validated LC-MS/MS method. The concentrations of the three analytes in both biological samples were determined and compared (Supplementary Figure S2). Pearson’s correlation analysis was performed to assess the relationship between seminal plasma and serum levels. The results showed that no significant correlation was observed for VE, 5-MTHF, or GSH between seminal plasma and serum, suggesting that the levels of these three antioxidants in seminal plasma were independent of their circulating concentrations in serum.

### 3.6 Validation and exploratory clinical association analysis of seminal VE, 5-MTHF, and GSH in infertile men

**FIGURE 5:**
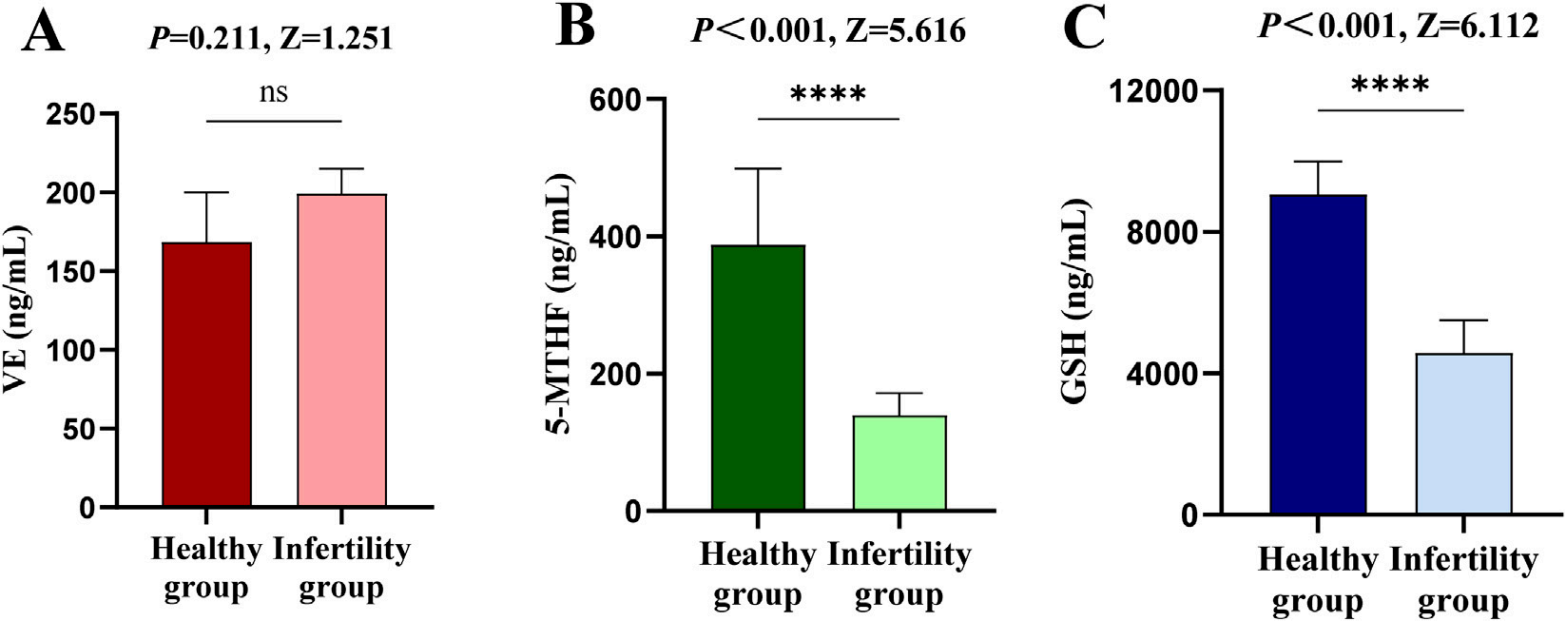
The levels of VE **(A)**, 5-MTHF **(B)** and GSH **(C)** between the healthy group and the infertility group.

To further evaluate the clinical applicability of the preliminary method-specific RIs, 47 infertile men were additionally included as a small validation cohort. Compared with the healthy group, the infertility group was older and showed significantly lower PR and total sperm motility (TSM), but higher DFI, while no significant differences were observed in most biochemical parameters (Table 7). The Mann–Whitney U test showed that seminal VE levels were not significantly different between the healthy and infertility groups [168.50 (129.00, 237.80) vs. 199.00 (138.00, 252.00) ng/mL]. In contrast, seminal 5-MTHF and GSH levels were significantly lower in the infertility group than in the healthy group [5-MTHF: 139.50 (68.02, 198.50) vs. 387.80 (152.50, 623.80) ng/mL; GSH: 4314.00 (3051.00, 5974.00) vs. 9049.00 (6128.00, 11,828.00) ng/mL] (Figure 5).

Considering the significant baseline differences in age and semen parameters between the two groups, multivariable linear regression models were further constructed to adjust for potential confounding factors using ln-transformed 5-MTHF and GSH as dependent variables, respectively. In the main age-adjusted model, group status remained significantly associated with seminal 5-MTHF and GSH levels. Additional sensitivity analyses further including PR, TSM, or DFI showed consistent results (Supplementary Table S9).

To explore the potential clinical value of seminal 5-MTHF and GSH, binary logistic regression models were established using infertility status as the dependent variable. In models adjusted for age, higher seminal 5-MTHF and GSH levels were both significantly associated with a lower probability of infertility. This association remained significant after further adjustment for PR, TSM, or DFI. Across the sensitivity models, the ORs for 5-MTHF were consistently less than 1, ranging from 0.312 to 0.994, with all *P* values <0.001. Similarly, the ORs for GSH ranged from 0.098 to 0.170 across the adjusted models, with all *P* values ≤0.001. These results suggested that reduced seminal 5-MTHF and GSH levels may be independently associated with infertility status (Supplementary Table S10).

Finally, linear regression analyses were performed to investigate the relationships between seminal VE, 5-MTHF, GSH, and semen parameters. No significant associations were observed between VE or 5-MTHF and semen volume, SC, PR, TSM, or DFI. For GSH, significant positive associations were observed with PR and TSM. Specifically, higher seminal GSH levels were associated with higher PR (β = 4.673, 95% CI: 0.416–8.930, *P* = 0.032) and higher TSM (β = 4.745, 95% CI: 0.189–9.301, *P* = 0.041). No significant associations were found between GSH and semen volume, SC, or DFI. These findings suggested that seminal GSH may be related to sperm motility parameters, whereas VE and 5-MTHF showed no obvious association with routine semen parameters in this exploratory analysis (Supplementary Figure S3).

## 4 Discussion

OS is closely involved in impaired sperm function and male infertility, partly through lipid peroxidation, disruption of redox homeostasis, and damage to sperm DNA (Bisht et al., 2017). In the present study, we developed and validated LC-MS/MS-based analytical procedures for the quantification of three non-enzymatic antioxidants, namely, VE, 5-MTHF, and GSH, in human seminal plasma. The method showed acceptable linearity, sensitivity, precision, accuracy, specificity, matrix effect, carryover, interference, and sample stability. Using this validated method, we further established preliminary method-specific RIs for these three analytes in healthy reproductive-aged Chinese men. These findings provide an analytical basis for future studies investigating local antioxidant status in seminal plasma.

In recent years, the use of LC-MS/MS for VE detection has been increasingly reported. The research focus in this field has centered primarily on the optimization of sample pretreatment methods, chromatographic separation, and the extensive application of stable isotope-labeled IS. However, to date, no mass spectrometry-based method has been reported for the quantification of VE in seminal plasma. Given that seminal plasma differs markedly from serum in lipid composition and matrix complexity, the validation of VE measurement with liquid-liquid extraction and VE-d6 in this matrix is an important methodological extension rather than a simple application of existing blood-based assays.

Research using LC-MS/MS for the quantification of 5-MTHF remains limited. According to Hyung et al. (Hyung et al., 2021), ascorbic acid was added during sample pretreatment to inhibit the oxidative degradation of 5-MTHF, under light-protected conditions throughout the process, and methanol was used as the organic solvent for methanol-mediated protein precipitation. Those were consistent with the treatment method in our study. The most notable difference between the two methodologies was observed in the sample purification step. Hyung et al. (Hyung et al., 2021) utilized a solid-phase extraction (SPE) column, which required sequential multi-step operations, including column activation, sample loading, washing, and gradient elution, ultimately resulting in prolonged processing time per sample and precluding the simultaneous handling of batch samples. In contrast, our study used a 96-well phospholipid removal plate for purification, which featured a simple operational procedure and high specificity. This strategy not only effectively eliminated cross-contamination between samples, but also enabled the targeted removal of high-content phospholipids—the primary interfering components in the seminal plasma matrix—thereby substantially reducing ion suppression effects in mass spectrometric detection.

Mass spectrometry-based detection of GSH in plasma or red blood cells has been widely studied globally. For example, Moore et al. (Moore et al., 2013) developed an LC-MS/MS method for measuring GSH and GSSG in whole blood, achieving one-step protein precipitation with sulfosalicylic acid (SSA) and derivatization with N-ethylmaleimide (NEM) to prevent artificial oxidation of GSH. Later, this approach was modified by Lee et al. (Lee et al., 2016) to treat mouse whole blood samples, separating protein precipitation and derivatization steps and using 0.1% trifluoroacetic acid (TFA) instead of FA as mobile phase A. Our research is consistent with these studies, employing SSA for protein precipitation to provide a clean analytical matrix. Moreover, stable isotope-labeled ISs were utilized to improve quantitative reliability by compensating for matrix-related ion suppression or enhancement. It must be noted that NEM derivatization was not performed in our method, simplifying sample processing, reducing non-specific reaction errors, and minimizing potential sample degradation or contamination. Because reduced GSH is susceptible to *ex vivo* oxidation, the absence of a derivatization step requires careful stabilization during sample handling. In this study, GSH oxidation was minimized by a combination of low-temperature processing, light protection, rapid seminal plasma separation, −80 °C storage, avoidance of repeated freeze–thaw cycles, and the use of an extraction reagent containing 5-sulfosalicylic acid, isopropanol, and ascorbic acid. 5-Sulfosalicylic acid enabled rapid protein precipitation and acidification of the sample matrix, thereby limiting enzymatic activity and oxidation-related changes, whereas ascorbic acid provided additional antioxidant protection during extraction. These procedures were designed to minimize GSH oxidation during sample processing, although derivatization was not performed. The following acceptable stability results after storage at −80 °C for 1 week, 1 month, and 6 months further supported the feasibility of this non-derivatized workflow for GSH quantification in seminal plasma.

The LC-MS/MS method can detect 5-MTHF and GSH in both serum and seminal plasma. Seminal plasma requires a 5-fold dilution to allow most clinical samples to fall within the linear calibration range and reduce viscosity. For samples exceeding the upper limit after routine dilution, additional dilution verification was performed. The maximum validated dilution factors extended the reportable ranges to 1.31–1645.75 ng/mL for 5-MTHF and 15.37–30730 ng/mL for GSH. The relative deviations after dilution remained within the acceptable range, supporting the reliability of quantification for high-concentration seminal plasma samples. The effect of dilution on low-concentration samples was also evaluated. In the clinical samples analyzed in this study, several 5-MTHF results were below 3 times LLMI after routine 5-fold dilution. These samples were reanalyzed without dilution, and the routine pre-dilution workflow did not introduce unacceptable quantitative bias near the lower measuring range (CLSI, 2014). For GSH, the observed concentrations in clinical seminal plasma samples were substantially higher than the LLMI, and therefore, additional low-concentration verification was not required. All deviations fell within the acceptable range, indicating that dilution procedures do not affect the quantitative accuracy of the two indicators.

Crucially, no consistent correlation was detected between serum and seminal plasma concentrations of VE, 5-MTHF, or GSH in the 22 paired samples. Several biological explanations may account for this observation. First, the blood-testis barrier creates a specialized microenvironment for germ cell development and restricts free exchange between the systemic circulation and the male reproductive tract (Babcock et al., 2026; Wanjari and Gopalakrishnan, 2024). Second, seminal plasma is not simply a filtrate of serum, but is composed of secretions from the testis, epididymis, prostate, and other accessory glands. Local synthesis, transport, and degradation may therefore influence antioxidant concentrations in seminal plasma independently of serum levels. Third, VE, 5-MTHF, and GSH may be differentially regulated by local OS, sperm metabolism, and accessory gland function. From a biomarker perspective, these findings indicate that serum measurements may not be suitable substitutes for seminal plasma measurements when evaluating the local reproductive redox environment. They also support the need for matrix-specific RIs and direct seminal plasma assessment in future biomarker studies. Future studies with larger paired cohorts are required to validate the biological and clinical significance of this compartmentalization.

In the small validation cohort of infertile men, seminal 5-MTHF and GSH levels were significantly lower than those in the healthy group, whereas VE levels did not differ significantly between groups. The lower seminal 5-MTHF level may reflect changes in local folate availability, methylation-related metabolism, or antioxidant defense capacity, while the lower GSH level may indicate reduced local redox buffering capacity. The lower seminal 5-MTHF level observed in infertile men deserves particular attention from the perspective of one-carbon metabolism. 5-MTHF is the biologically active form of folate and serves as a key methyl donor for DNA methylation reactions. In male reproduction, adequate one-carbon metabolism is important for spermatogenesis, sperm DNA integrity, chromatin remodeling, epigenetic regulation, and early embryonic developmental potential. Therefore, reduced seminal 5-MTHF may indicate not only weakened local antioxidant capacity, but also impairment of the folate-dependent one-carbon metabolic and methylation microenvironment in the male reproductive tract. To further evaluate the robustness of the group comparison, regression models were performed. Age was included as a confounder in the main model because the infertility group was older than the healthy group, and age may influence reproductive function and oxidative status. In contrast, PR, TSM, and DFI were not included together as main-model confounders because they are likely to represent manifestations of infertility status and may even be downstream consequences of impaired antioxidant status. Including them simultaneously in the primary model could lead to overadjustment and obscure biologically meaningful associations. Therefore, PR, TSM, and DFI were considered only in sensitivity analyses. The associations between infertility status and lower seminal 5-MTHF and GSH levels remained generally consistent after additional adjustment for these semen parameters, supporting the stability of the exploratory findings.

This study has several limitations. First, the RIs were established based on a single-center cohort of Chinese men of reproductive age. Because antioxidant levels in seminal plasma may be influenced by ethnicity, geographic region, lifestyle, environmental exposure, and laboratory workflow, the present RIs should be considered population- and method-specific. The generalizability to other ethnic groups, regions, and laboratories remains uncertain. Second, although 120 healthy men met the minimum sample size recommended for non-parametric RI estimation, the sample size was still limited for subgroup analyses or stratified RIs. Larger studies are needed to determine whether age, body mass index (BMI), lifestyle, metabolic status, or semen characteristics influence these analytes. Third, the infertile cohort was relatively small and heterogeneous, and the clinical association analysis was exploratory. Therefore, the observed differences in 5-MTHF and GSH between the healthy and infertile groups require external validation. Future validation should include patients from different clinical centers and infertile men with different etiologies, disease severities, and semen parameter abnormalities, to determine whether the associations observed in this exploratory cohort are reproducible and clinically meaningful. Fourth, the current workflow does not allow simultaneous high-throughput detection of all three indicators from a single sample aliquot due to the distinct physicochemical properties and stability requirements of each. In future work, we aim to recruit larger and more diverse populations and conduct multicenter validation or inter-laboratory reproducibility assessment. Further still, we can combine routine semen parameters with other OS biomarkers to construct models of infertility and explore the clinical value of VE, 5-MTHF, and GSH in evaluating male reproductive oxidative status.

In conclusion, this study developed and validated LC-MS/MS-based methods for the quantification of VE, 5-MTHF, and GSH in seminal plasma and established preliminary method-specific RIs in healthy Chinese men. In a small exploratory cohort, lower seminal 5-MTHF and GSH levels were observed in the infertile group, whereas VE levels did not differ significantly between groups. These findings suggest that the validated method may serve as a promising exploratory approach for future studies of seminal antioxidant status and male infertility, but further multicenter validation and clinical studies are required before routine clinical application can be recommended.

## Data Availability

The raw data supporting the conclusions of this article will be made available by the authors, without undue reservation.

## References

[B1] Adeoye O. Olawumi J. Opeyemi A. Christiania O. (2018). Review on the role of glutathione on oxidative stress and infertility. JBRA Assist. Reprod. 22, 61–66. 10.5935/1518-0557.20180003 29266896 PMC5844662

[B2] Author anonymous (2024). Focusing on male infertility. Nat. Rev. Urol. 21 (2), 61. 10.1038/s41585-024-00856-0 38228741

[B3] Babcock R. L. Haynes A. Dufour J. M. (2026). Testicular immune privilege revisited: when protection fails. Fertil. Steril. 125 (5), 737–745. 10.1016/j.fertnstert.2026.03.018 41856349

[B4] Bisht S. Faiq M. Tolahunase M. Dada R. (2017). Oxidative stress and Male infertility. Nat. Rev. Urol. 14, 470–485. 10.1038/nrurol.2017.69 28508879

[B5] Boxmeer J. C. Smit M. Utomo E. Romijn J. C. Eijkemans M. J. C. Lindemans J. (2009). Low folate in seminal plasma is associated with increased sperm DNA damage. Fertil. Steril. 92, 548–556. 10.1016/j.fertnstert.2008.06.010 18722602

[B6] Brannigan R. E. Hermanson L. Kaczmarek J. Kim S. K. Kirkby E. Tanrikut C. (2024). Updates to Male infertility: AUA/ASRM guideline (2024). J. Urol. 212 (6), 789–799. 10.1097/JU.0000000000004180 39145501

[B7] CLSI (2010). Defining, Establishing, and Verifying Reference Intervals in the Clinical Laboratory (EP28-A3c). Available online at: https://clsi.org/shop/standards/ep28/ .

[B8] CLSI (2007). Prelimin in the Clinical Laboratory: General Principles and Guidance; Approved Guideline. CLSI Document C50-A. Wayne, PA: Clinical and Laboratory Standards Institute.

[B9] CLSI (2014). Liquid chromatography-mass Spectrometry Methods; Approved Guideline. CLSI Document C62-A. Wayne, PA: CLSI.

[B10] Eisenberg M. L. Esteves S. C. Lamb D. J. Hotaling J. M. Giwercman A. Hwang K. (2023). Male infertility. Nat. Rev. Dis. Prim. 9 (1), 49. 10.1038/s41572-023-00459-w 37709866

[B11] El-Khoury J. M. Bunch D. R. Reineks E. Jackson R. Steinle R. Wang S. (2012). A simple and fast liquid chromatography-tandem mass spectrometry method for measurement of underivatized L-arginine, symmetric dimethylarginine, and asymmetric dimethylarginine and establishment of the reference ranges. Anal. Bioanal. Chem. 402 (2), 771–779. 10.1007/s00216-011-5462-9 22124751

[B12] Evans E. P. P. Scholten J. T. M. Mzyk A. Reyes-San-Martin C. Llumbet A. E. Hamoh T. (2021). Male subfertility and oxidative stress. Redox Biol. 46, 102071. 10.1016/j.redox.2021.102071 34340027 PMC8342954

[B13] Fang Y. Su Y. Xu J. Hu Z. Zhao K. Liu C. (2021). Varicocele-Mediated Male infertility: from the perspective of testicular immunity and inflammation. Front. Immunol. 12, 729539. 10.3389/fimmu.2021.729539 34531872 PMC8438154

[B14] Fazili Z. Whitehead R. D. Jr Paladugula N. Pfeiffer C. M. (2013). A high-throughput LC-MS/MS method suitable for population biomonitoring measures five serum folate vitamers and one oxidation product. Anal. Bioanal. Chem. 405 (13), 4549–4560. 10.1007/s00216-013-6854-9 23462981 PMC5326548

[B15] Ferrazzi E. Tiso G. Di Martino D. (2020). Folic acid versus 5- methyl tetrahydrofolate supplementation in pregnancy. Eur. J. Obstet. Gynecol. Reprod. Biol. 253, 312–319. 10.1016/j.ejogrb.2020.06.012 32868164

[B16] Hamilton Lauren E. Oko R. Miranda-Vizuete A. Sutovsky P. (2022). Sperm redox system equilibrium: implications for fertilization and male fertility. Adv. Exp. Med. Biol. 1358, 345–367. 10.1007/978-3-030-89340-8_15 35641877

[B17] Hyung S.-W. Lee S. Han J. Lee J. Beak S.-Y. Kim B. (2021). Highly sensitive analytical method for the accurate determination of 5-methyltetrahydrofolic acid monoglutamate in various volumes of human plasma using isotope dilution ultra-high performance liquid chromatography-mass spectrometry. J. Chromatogr. B Anal. Technol. Biomed. Life Sci. 1179, 122725. 10.1016/j.jchromb.2021.122725 34311437

[B18] Lamb D. J. (2025). Chromosome defects and male factor infertility. Fertil. Steril. 123 (6), 933–942. 10.1016/j.fertnstert.2025.04.034 40311997 PMC13508826

[B19] Le J. Yuan T. F. Zhang Y. Wang S. T. Li Y. (2018). New LC-MS/MS method with single-step pretreatment analyzes fat-soluble vitamins in plasma and amniotic fluid. J. Lipid Res. 59 (9), 1783–1790. 10.1194/jlr.D087569 30026263 PMC6121937

[B20] Lee S.-G. Yim J. Lim Y. Kim J.-H. (2016). Validation of a liquid chromatography tandem mass spectrometry method to measure oxidized and reduced forms of glutathione in whole blood and verification in a mouse model as an indicator of oxidative stress. J. Chromatogr. B Anal. Technol. Biomed. Life Sci. 1019, 45–50. 10.1016/j.jchromb.2015.10.041 26575459

[B21] Li S. W. Luo Y. Chen W. Huang C. Chen Y. Hong X. Y. (2026). A novel liquid chromatography-tandem mass spectrometry (LC-MS/MS) method for quick and efficient quantification of five fat-soluble vitamins. Clin. Chim. Acta 579, 120615. 10.1016/j.cca.2025.120615 40967265

[B22] Liu Z. Jin L. Zhang J. Zhou W. Zeng J. Zhang T. (2022). The establishment of LC-MS/MS assays-specific reference intervals for serum folates and its application in evaluating FA-supplemented folate deficiency patients: appeals for a suitable and individualized supplementation. Clin. Chim. Acta 537, 96–104. 10.1016/j.cca.2022.09.019 36261072

[B23] Maxones A. Beck E. Rimbach G. Birringer M. (2024). A new LC-MS/MS-Based method for the simultaneous detection of α-Tocopherol and its long-chain metabolites in plasma samples using stable isotope dilution analysis. Pharm. (Basel) 17 (11), 1405. 10.3390/ph17111405 39598322 PMC11597593

[B24] Minhas S. Boeri L. Capogrosso P. Cocci A. Corona G. Dinkelman-Smit M. (2025). European Association of Urology Guidelines on Male sexual and reproductive health: 2025 update on Male infertility. Eur. Urol. 87 (5), 601–616. 10.1016/j.eururo.2025.02.026 40118737

[B25] Moore T. Le A. Niemi A.-K. Kwan T. Cusmano-Ozog K. Enns G. M. (2013). A new lc–ms/Ms method for the clinical determination of reduced and oxidized glutathione from whole blood. J. Chromatogr. B Anal. Technol. Biomed. Life Sci. 929, 51–55. 10.1016/j.jchromb.2013.04.004 23660247

[B26] Ribeiro J. C. Nogueira-Ferreira R. Amado F. Alves M. G. Ferreira R. Oliveira P. F. (2022). Exploring the role of oxidative stress in sperm motility: a proteomic network approach. Antioxid. Redox Signal 37 (7-9), 501–520. 10.1089/ars.2021.0241 34847748

[B27] Sharma A. Minhas S. Dhillo W. S. Jayasena C. N. (2021). Male infertility due to testicular disorders. J. Clin. Endocrinol. Metab. 106 (2), e442–e459. 10.1210/clinem/dgaa781 33295608 PMC7823320

[B28] Škovierová H. Vidomanová E. Mahmood S. Sopková J. Drgová A. Červeňová T. (2016). The molecular and cellular effect of homocysteine metabolism imbalance on human health. Int. J. Mol. Sci. 17, 1733. 10.3390/ijms17101733 27775595 PMC5085763

[B29] Su L. Qu H. Cao Y. Zhu J. Zhang S. Z. Wu J. (2022). Effect of antioxidants on sperm quality parameters in subfertile men: a systematic review and network meta-analysis of randomized controlled trials. Adv. Nutr. 13 (2), 586–594. 10.1093/advances/nmab127 34694345 PMC8970840

[B30] Wang W. Xu W. Qiao S. Xu H. Ji E. Liu T. (2026). Development, validation, and four-year clinical application of a novel, rapid, sensitive, and high-throughput UPLCMS/MS method for the simultaneous determination of vitamins A and E in 50 μL of serum. J. Pharm. Biomed. Anal. 269, 117237. 10.1016/j.jpba.2025.117237 41218421

[B31] Wanjari U. R. Gopalakrishnan A. V. (2024). Blood-testis barrier: a review on regulators in maintaining cell junction integrity between sertoli cells. Cell Tissue Res. 396 (2), 157–175. 10.1007/s00441-024-03894-7 38564020

[B32] World Health Organization (2010). WHO Laboratory Manual for the Examination and Processing of Human Semen. 5th ed. Geneva: World Health Organization.

[B33] Wright C. Milne S. Leeson H. (2014). Sperm DNA damage caused by oxidative stress: modifiable clinical, lifestyle and nutritional factors in male infertility. Reprod. Biomed. Online 28 (6), 684–703. 10.1016/j.rbmo.2014.02.004 24745838

[B34] Zhou X. Shi H. Zhu S. Wang H. Sun S. (2022). Effects of vitamin E and vitamin C on male infertility: a meta-analysis. Int. Urol. Nephrol. 54 (8), 1793–1805. 10.1007/s11255-022-03237-x 35604582

